# African swine fever virus infection activates inflammatory responses through downregulation of the anti-inflammatory molecule C1QTNF3

**DOI:** 10.3389/fimmu.2022.1002616

**Published:** 2022-10-12

**Authors:** Changjie Lv, Qiang Zhang, Li Zhao, Jingyu Yang, Zhong Zou, Ya Zhao, Chengfei Li, Xiaomei Sun, Xian Lin, Meilin Jin

**Affiliations:** ^1^ College of Veterinary Medicine, Huazhong Agricultural University, Wuhan, China; ^2^ New-onset department, Research Institute of Wuhan Keqian Biology Co., Ltd, Wuhan, China; ^3^ Department of pig disease prevention and control, The Cooperative Innovation Center for Sustainable Pig Production, Wuhan, China; ^4^ College of Biomedicine and Health, Huazhong Agricultural University, Wuhan, China; ^5^ State Key Laboratory of Biocatalysis and Enzyme Engineering, School of Life Sciences, Hubei University, Wuhan, China; ^6^ CAS Key Laboratory of Special Pathogens and Biosafety, Wuhan Institute of Virology, Center for Biosafety Mega-Science, Chinese Academy of Sciences, Wuhan, China

**Keywords:** African swine fever virus, quantitative proteomics, C1QTNF3, inflammatory process, host-virus infection

## Abstract

African swine fever (ASF) is the most dangerous pig disease, and causes enormous economic losses in the global pig industry. However, the mechanisms of ASF virus (ASFV) infection remains largely unclear. Hence, this study investigated the host response mechanisms to ASFV infection. We analyzed the differentially expressed proteins (DEPs) between serum samples from ASFV-infected and uninfected pigs using quantitative proteomics. Setting the *p*-value < 0.05 and |log_2_ (fold change)| > 1.5, we identified 173 DEPs, comprising 57 upregulated and 116 downregulated proteins, which belonged to various biological processes and pathways based on the Gene Ontology and Kyoto Encyclopedia of Genes and Genomes pathway enrichment analyses. The enriched pathways include immune responses, metabolism, and inflammation signaling pathways. Western blot analysis validated the DEPs identified using quantitative proteomics. Furthermore, our proteomics data showed that C1QTNF3 regulated the inflammatory signaling pathway. C1QTNF3 knockdown led to the upregulation of pro-inflammatory factors IL-1β, IL-8, and IL-6, thus inhibiting ASFV replication. These results indicated that C1QTNF3 was critical for ASFV infection. In conclusion, this study revealed the molecular mechanisms underlying the host-ASFV interaction, which may contribute to the development of novel antiviral strategies against ASFV infection in the future.

## Introduction

African swine fever (ASF) is one of the most severe infectious diseases of pigs worldwide with its virulent strain mortality rate of up to 100% (1, 2), and causes enormous economic loss. ASF virus (ASFV) is the only known DNA arbovirus, and it is a sole member of the *Asfarviridae* family and *Asfivirus* genus (3). ASFV contains a 170–190 kb genome that encodes 151–167 genes depending on the strain (4). ASFVs are divided into 24 genotypes based on the B646L gene-encoding capsid protein p72 (5). To date, there have been no effective vaccines or antiviral drugs to control ASFVs in domesticated pigs and wild boars (6, 7). Currently, the prevention and control of ASF mainly depend on rigorous import policies and biosecurity measures, including disinfection, culling of infected animals, and early detection.

ASF was first reported in Kenya in 1921, followed by its discovery in other African countries. ASF was reported in Europe in the 1950s (8). Later in 2007, there was an outbreak of genotype II ASFV in Georgia and Armenia. In China, ASF was first discovered in 2018, followed by outbreaks in Mongolia and Vietnam in 2019 (9). In recent years, various naturally-mutated, more transmissible, and more virulent ASFV strains have emerged, which poses a great challenge to the prevention and control of the virus (10, 11). Therefore, it is necessary to elucidate the host–virus interaction mechanisms of ASFV. A genome-wide transcriptomic analysis of ASFV-infected primary porcine alveolar macrophages (PAMs) revealed that multiple pathways are involved in the responses against ASFV, including the host immune response and metabolic processes (12). Proteomics of ASFV-infected PAMs also show that immune system response, complement and coagulation cascade, and metabolic processes are crucial pathways during the infection (13). Furthermore, transcriptomics of the different tissues infected by ASFV can reveal the activation of their homologous pathways (14). Although such studies have found numerous critical proteins and pathways, the molecular mechanisms of host-virus interaction remain largely unknown.

Quantitative proteomic analysis of serum samples has been used to investigate the host response to several viruses, including the chikungunya virus, human immunodeficiency virus, porcine epidemic diarrhea virus, and classical swine fever virus. Studies revealed that antiviral factors and immune- and inflammation-related pathways are vital for the survival or protection of hosts (15–18). The proteomic analysis of serum samples may contribute to exploring the changes in host proteins during viral infection. Therefore, this study aimed to reveal the host-virus interaction mechanisms after ASFV infection using quantitative proteomic analysis of ASFV-infected serum samples.

We analyzed the quantifiable proteins from the serum samples of ASFV-infected pigs using tandem mass tagging (TMT)-based quantitative proteomics. Comparing infected with uninfected samples, we found that the differentially expressed proteins (DEPs) were mainly enriched in the immune-, inflammation-, and metabolism-related pathways. Of these altered proteins, we focused on inflammation-related C1Q and tumor necrosis factor-related protein 3 (C1QTNF3, also called CORS26, CTRP3, or cartducin), which is a member of the C1q/TNF-related protein-family (19, 20). C1QTNF3 has four domains, namely the N-terminal signal peptide, C-terminal C1Q globular domain, variable domain, and collagen repeat (20). Of these four domains, C1QTNF3 exerts functions mainly based on the C1Q globular domain. C1QTNF3 plays an important regulatory role in cartilage development, inflammatory response, cardiovascular disease, adipocyte differentiation, and metabolism (21–24). C1QTNF3 inhibits LPS-induced inflammation *via* PPAR-γ and TLR4-mediated pathways (25). ASFV infection also induces the expression of inflammatory cytokines through the TLRs/MyD88 pathway (26). C1QTNF3 might affect ASFV proliferation through the inflammatory signaling pathway. In addition, we confirmed that the downregulation of C1QTNF3 by siRNA increased ASFV-induced inflammatory cytokine production in PAMs, thereby inhibiting ASFV proliferation. Our findings provide the first insights into the host response to ASFV infection using serum samples, and lay a solid foundation for further research on the host-virus interaction of ASFV.

## Materials and methods

### Cells, viruses, and serum samples

PAMs were obtained from 5-week-old specific pathogen-free pigs using bronchoalveolar lavage as previously described (27). Porcine kidney-15 (PK-15) cells were obtained from our laboratory. PAMs and PK-15 cells were grown in RPMI 1640 culture medium (Gibco, USA) and Dulbecco’s modified Eagle medium (Gibco, USA), respectively, supplemented with 10% fetal bovine serum (Gibco, USA), 100 μg/mL streptomycin, and 100 IU/mL penicillin at 37°C in 5% CO_2_. The virulent genotype II ASFV was previously isolated from wild boar and kept in a biosafety level 3 laboratory for *in vitro* experiments, and the genome has been uploaded to the National Center for Biotechnology Information database (GenBank: OM161110). The virus was proliferated in PAMs and stored at –80°C until used. Swine serum samples of ASFV-infected and uninfected pigs from the same pig farm were stored in our laboratory.

### Reagents and antibodies

The recombinant porcine IL-1β and IL-6 proteins were purchased from Sino Biological (China), the recombinant porcine IL-8 proteins were purchased from Abcam (UK), and dsDNA naked Poly (dA:dT) was purchased from *In vivo*Gen (USA). The primary antibodies used in this study were rabbit anti-PSMC3 (1:1000, Cell Signaling Technology, USA), rabbit anti-SLC38A2 (1:1000, Invitrogen, USA), rabbit anti-EEF1G (1:1000, Invitrogen, USA), rabbit anti-MMP8 (1:1000, Cell Signaling Technology, USA), rabbit anti-C1QTNF3 (1:1500, Bioss, China), and mouse anti-GAPDH (1:8000, Proteintech, China). The secondary antibodies used were horseradish peroxidase-conjugated goat anti-rabbit IgG (1:10000, Proteintech, China) and goat anti-mouse IgG (1:10000, Proteintech, China).

### Protein extraction and trypsin digestion

The cellular debris and impurities in the 100-μL serum sample were removed using centrifugation at 4 °C at 12,000 × *g* for 10 min. Subsequently, in a new 1.5-mL centrifuge tube, the supernatant was transferred and the protein concentration was determined with the PierceTM BCA protein assay kit (Thermo Fisher Scientific, USA), according to the manufacturer’s instructions.

For trypsin digestion, the protein solution was deoxidized with 5 mM dithiothreitol at 56 °C for 30 min, and alkylated with 11 mM iodoacetamide for 15 min in the dark at 37°C. Then, the protein sample was diluted by adding 100 mM tetraethylammonium bromide, leading to the final concentration of urea not being more than 2 M. Finally, trypsin was added at 1:50 mass ratio overnight for the first digestion, followed by the secondary digestion at 1:100 mass ratio for 4 h.

### TMT labeling

The different groups of peptides produced after trypsin digestion were labeled using the TMT kit (Thermo Fisher, USA), according to the manufacturer’s instructions. Briefly, the TMT reagent was thawed and dissolved in acetonitrile. The peptide mixtures were then incubated for 2 h at 37°C, desalted, and dried using vacuum centrifugation.

### LC-MS/MS analysis

The labeled peptides were fractionated using high pH reverse-phase high-performance liquid chromatography with the Thermo Betasil C18 column (10 mm ID, 5 μm particles, and 250 mm in length), according to the manufacturer’s instructions. The tryptic peptides in 0.1% formic acid (solvent A) were dissolved and directly loaded onto a homemade reversed-phase analytical column (75 μm, 15 cm length) (Thermo Fisher Scientific, USA). The gradient comprised of an increasing percentage of solvent B (0.1% formic acid in 98% acetonitrile) over 26 min from 6%–23%, 23%–35% in the next 12 min, 35%–80% in the next 3 min, and then kept at 80% for the last 3 min; all at a constant flow rate of 400 nL/min on an EASY-nLC 1000 ultra-performance liquid chromatography (UPLC) system (Thermo Fisher, USA).

The resulting peptides were subjected to a nanospray ionization source followed by tandem mass spectrometry (MS/MS) using the Q Exactive™ Plus Orbitrap mass spectrometer (Thermo Fisher, USA) coupled online to the UPLC system. The electrospray voltage was set as 2.0 kV. A 350–1800 m/z MS was used for the entire scan range. The Orbitrap was used to detect intact peptides at a resolution of 70,000. We then selected peptides for MS/MS using 28% normalized collision energy, and fragments were detected in the Orbitrap at a resolution of 17,500.The automatic gain control was set at 5E4 for MS/MS and the fixed first mass was set as 100 m/z. A data-dependent procedure alternated between one MS scan and 20 MS/MS scans with a dynamic exclusion time of 15 s.

### Database search

The MS/MS data were analyzed using the Maxquant software (version 1.5.2.8). Concatenated with the reverse decoy database, the tandem mass spectra were searched against the UniProt database of *Sus scrofa* (pig). Trypsin/P was specified as the cleavage enzyme allowing up to two missing cleavages. For the initial search, the mass tolerance was set as 20 ppm for precursor ions and 5 ppm in the mass search, and the mass tolerance for fragment ions was set as 0.02 Da. Oxidation of methionine was specified as a variable modification and the modification of cysteine with carbamidomethyl was specified as a fixed modification. The false discovery rate was modulated to < 1% and the minimum score for modified peptides was set as > 40.

### Functional analysis of the differentially expressed proteins

To further explore the internal relationships between the DEPs, an enrichment analysis was performed. The Gene Ontology (GO) annotation was analyzed from the UniProt-GOA database (http://www.ebi.ac.uk/GOA/) for three categories—biological process, molecular function, and cellular component. The Clusters of Orthologous Groups of protein/Eukaryotic Orthologous Groups of proteins (COG/KOG) analysis was performed using an online database (http://ftp.ncbi.nih.gov/pub/COG/KOG/kyva).

To confirm the enrichment of DEPs, the two-tailed Fisher’s exact test was used to identify enriched pathways in the Kyoto Encyclopedia of Genes and Genomes (KEGG). The whole quantified protein annotations were used as background data.

### Western blot analysis

For western blot analysis, the proteins of cells or serum samples were separated using sodium dodecyl sulfate-polyacrylamide gel electrophoresis and transferred onto a nitrocellulose filter membrane (GE Technology, USA) in the transfer buffer. The membrane was blocked with 1% bovine serum albumin at 37°C for 1 h. Subsequently, the membrane was incubated with a primary antibody at 37°C for 2 h followed by three washes with tris buffered saline plus Tween 20 (TBST). Finally, the membrane was incubated with the corresponding secondary antibody at 37°C for 1 h and washed three times with TBST. The specific bands on the membrane were visualized using the western blot ECL reagent (Advansta, USA).

### Enzyme-linked immunosorbent assay (ELISA)

A commercially available ASFV VP72 antibody blocking ELISA kit (INgezim 11.PPA.K3, Madrid, Spain) was used to detect antibodies of six serum samples. Cell supernatants were collected, and porcine IL-6, IL-1β, and IL-8 were detected using porcine IL-6, IL-1β, and IL-8 ELISA kit (Solarbio). The measured value was calculated based on a standard curve according to the manufacturer’s instructions.

### Real-time quantitative PCR

To assess the change in cytokines of PAMs during ASFV infection, the real-time quantitative polymerase chain reaction (RT-qPCR) was performed using the primers listed in **Table S1**. Briefly, the total RNA of PAMs was extracted using an RNA extraction kit (Magen, Guangzhou, China), followed by reverse transcription to cDNA using HiScript Reverse Transcriptase (Vazyme, Nanjing, China). PCRs were conducted in a QuantStudio™ 6 Flex system (Life Technologies, USA) as follows: 95°C for 10 min, 40 cycles of 95°C for 15 s, 60°C for 15 s, and 72°C for 15 s. The 2^-ΔΔCt^ quantification method was used to analyze the qPCR results.

### Detection of viral titer

We performed the hemadsorption (HAD) assay to detect the titer of ASFV as previously described (28), with minor modifications. Briefly, PAMs were seeded in 96-well plates. The virus samples were diluted in a continuous 10-fold dilution and then pipetted into the wells of the 96-well plates with eight replicates in each gradient. Next, 30 µL of 1% swine red blood cells was added to each well after 3 days of cultivation, and a rosette formation representing hemadsorption was observed around the infected cells. The hemadsorption was observed until 7 days postinfection and 50% HAD dose (HAD_50_) was calculated using the Reed–Muench method (29).

### Transfection of siRNAs and plasmid

The siRNA of porcine C1QTNF3 or the negative control was synthesized by JTS scientific (Wuhan, China). The compositive siRNA was dissolved in sterile diethylpyrocarbonate-treated water and transfected into PAMs at a final concentration of 60 nM, using the Lipofectamine™ RNAiMAX Transfection Reagent (Thermo Fisher, USA), according to the manufacturer’s instructions. The siRNA sequences are listed in **Table S2**.

The full-length sequence of the porcine C1QTNF3 was cloned into a pCAGGS-HA vector to construct the recombinant plasmid C1QTNF3-HA. The constructed plasmid was analyzed and verified using DNA sequencing. PAMs and PK-15 cells were transfected with the recombinant plasmid C1QTNF3-HA using the jetPRIME^®^ transfection reagent (Polyplus-transfection, France) according to the manufacturer’s instructions.

### Biosafety statement

All live ASFV experiments were performed in an animal biosafety level 3 (ABSL-3) laboratory according to the relevant laws and regulations.

### Statistical analysis

All data were analyzed for statistical significance using the Student’s *t*-test. Significant differences were affirmed when *p* < 0.05 (*), *p* < 0.01 (**), and *p* < 0.001 (***).

## Results

### DEPs associated with ASFV infection

**Figure 1:**
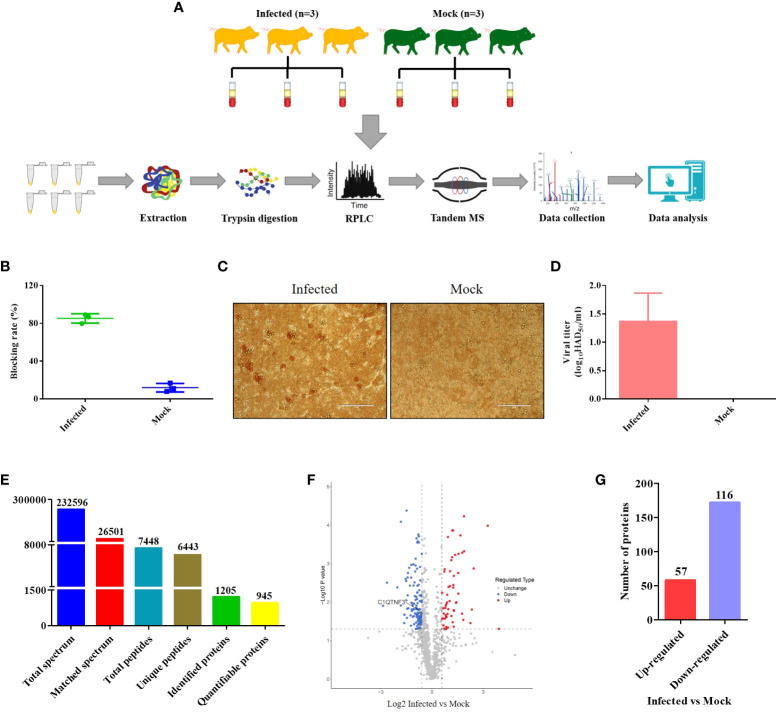
Schematic of the experimental method and data on the differentially expressed proteins (DEPs) identified between the ASFV-infected and uninfected serum samples using proteomics. **(A)** The serum samples of three ASFV-infected and uninfected pigs were collected from the same pig farm and subjected to quantitative proteomics through tandem mass spectrometry technology, high-performance liquid chromatography, and mass spectrometry. **(B)** Antibody, **(C, D)** Titer of the African swine fever virus was measured between the ASFV-infected and uninfected serum samples using ELISA and the HAD assay, respectively. **(E)** Summary of the quantitative proteomics data of swine serum samples. **(F)** Volcano map of DEPs between the ASFV-infected and uninfected serum samples. **(G)** Number of DEPs between the ASFV-infected and uninfected serum samples.

The schematic of the experimental workflow is shown in **Figure 1A**. Serum samples of healthy pigs (n = 3) and ASFV-infected pigs (n = 3) were collected from the same pig farm. Pigs infected with ASFV rather than healthy pigs showed symptoms of the disease, including staggering gait, anorexia, depression, and cough. The pigs were approximately 110 days old. Six serum samples (one from each pig) were analyzed using the OIE-recommended INgezim ASFV antibody blocking ELISA kit according to the manufacturer’s instructions, and three negative and three positive results were obtained (**Figure 1B**). The viral titers of the six serum samples were also analyzed using the HAD assay (**Figure 1C**). The viral titers of the positive serum samples were less than 10^2^ HAD_50_/mL (**Figure 1D**), which is consistent with a previous report that viral titers were less than 10^2^ HAD_50_/mL in the blood in the early stage of infection (30). The proteomic analysis yielded 232,596 raw data (**Figure 1E**). The raw spectra were further searched in the protein database, and 26,501 clean spectra were obtained (**Figure 1E**). Ultimately, 945 quantifiable proteins were mapped to the pig reference genome. Based on the thresholds of *p*-value < 0.05 and |log_2_ (fold change)| > 1.5, 173 DEPs were identified from the swine serum samples by comparing the ASFV versus non-ASFV infection groups, of which 57 were upregulated and 116 were downregulated DEPs (**Figures 1F, G**). C1QTNF3 is marked in **Figure 1F**. The identified DEPs are presented in **Supplementary Table S3**.

### Enrichment analysis of DEPs

**Figure 2:**
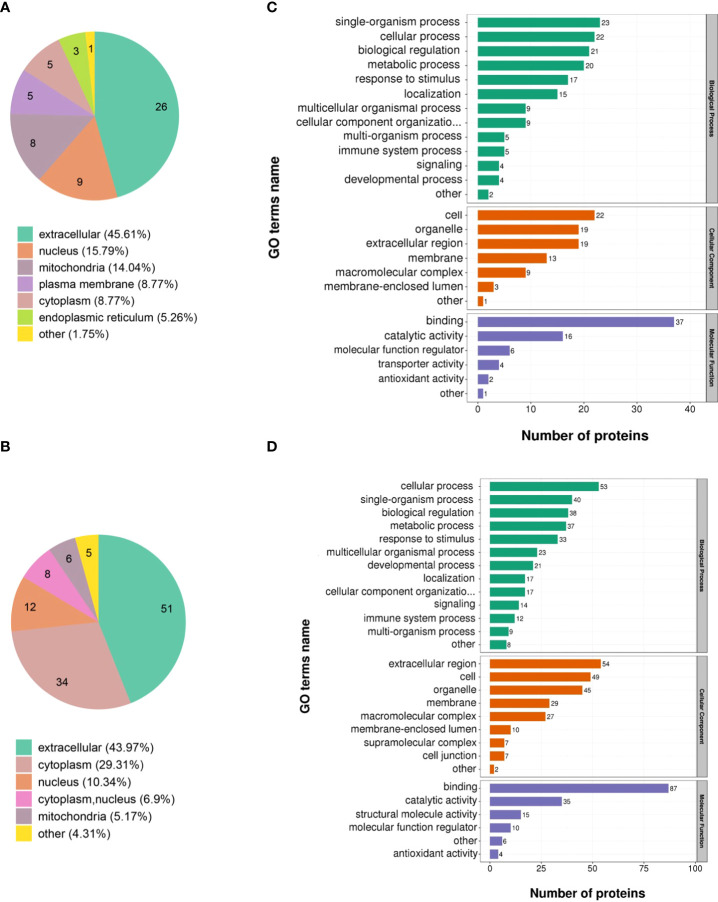
Functional analysis of the DEPs identified through quantitative proteomics in ASFV-infected versus uninfected samples. Subcellular structural analysis of **(A)** upregulated DEPs and **(B)** downregulated DEPs. GO enrichment analysis of **(C)** upregulated DEPs and **(D)** downregulated DEPs.

The 173 DEPs were functionally annotated using GO enrichment, KEGG pathway analysis, and COG/KOG analysis. The subcellular localization results showed that the largest number of the upregulated (45.61%) and downregulated proteins (43.97%) were distributed in the extracellular matrix (**Figures 2A, B**). In addition, 15.79% of the upregulated proteins were located in the nucleus and 29.31% of the downregulated proteins were located in the cytoplasm (**Figures 2A, B**).

GO annotation categorized the 173 DEPs based on biological processes, cellular components, and molecular function. The upregulated proteins were classified into 26 functional groups. Among them, biological processes possessed 13 GO terms, and the most representative were “single-organism process” and “cellular process.” Cellular components possessed 7 GO terms, and the most representative were “cell” and “organelle.” Moreover, molecular function possessed 6 GO terms, and the most representative terms were “binding” and “catalytic activity” (**Figure 2C**). Furthermore, the downregulated proteins were annotated to 28 GO terms. Among them, 13 GO terms represented biological processes, and the most representative were “cellular process” and “single-organism process.” The 9 and 6 GO terms belonged to the cellular component and molecular function, respectively, and the most representative cellular component GO terms were “extracellular region” and “cell” while the most representative molecular function GO terms were “binding” and “catalytic activity” (**Figure 2D**).

**Figure 3:**
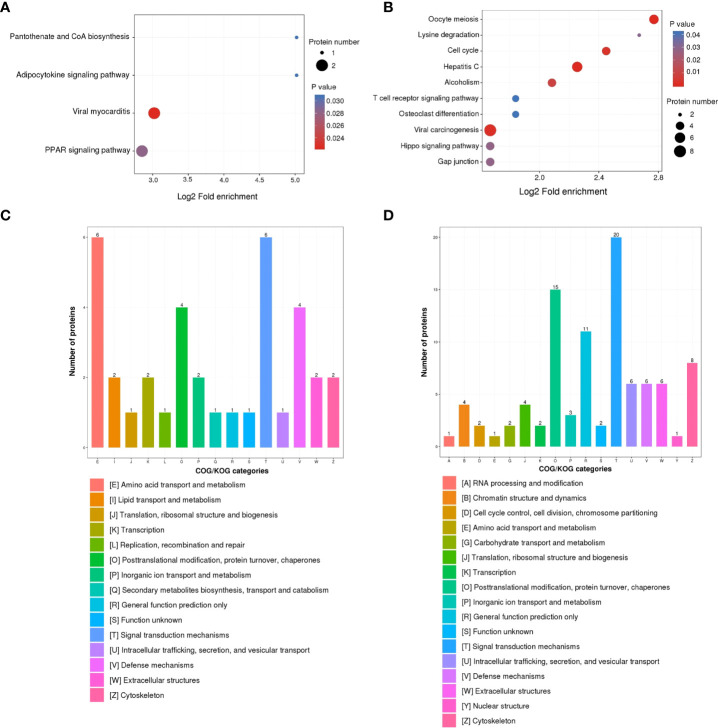
KEGG pathway and COG/KOG functional classification analysis of DEPs in ASFV-infected versus uninfected samples using quantitative proteomics. KEGG pathway analysis of **(A)** upregulated DEPs and **(B)** downregulated DEPs. COG/KOG functional classification analysis of **(C)** upregulated DEPs and **(D)** downregulated DEPs.

The KEGG pathway enrichment analysis was used based on the upregulated and downregulated proteins. The results show that the upregulated proteins were enriched in the pantothenate and CoA biosynthesis, adipocytokine signaling pathway, viral myocarditis, and PPAR signaling pathway (**Figure 3A**). Correspondingly, the downregulated proteins were enriched in oocyte meiosis, lysine degradation, cell cycle, hepatitis C infection, alcoholism, T cell receptor signaling pathway, osteoclast differentiation, viral carcinogenesis, Hippo signaling pathway, and gap junction (**Figure 3B**).

To better analyze the DEPs, the COG/KOG analysis was applied. Among the upregulated proteins, the largest proportion of proteins belonged to the categories of amino acid transport, metabolism, and signal transduction mechanisms; followed by posttranslational modification, protein turnover, chaperones, and defense mechanisms (**Figure 3C**). Furthermore, the downregulated proteins were in the categories of signal transduction mechanisms, posttranslational modification, protein turnover, and chaperones (**Figure 3D**).

Western blot analysis was used to validate some of the DEPs identified in the LC-MS/MS analysis. To more accurately evaluate the results of LC-MS/MS, we did not choose proteins with the highest or lowest levels, but randomly selected DEPs—PSMC3, SLC38A2, EEF1G, MMP8, and C1QTNF3 (**Figure S1**). We found that the levels of PSMC3 and SLC38A2 were higher, whereas the levels of EEF1G, MMP8, and C1QTNF3 were lower in the ASFV-infected serum samples than in the mock group, which was consistent with the results of the quantitative proteomics, indicating the reliability of the LC-MS/MS results.

We also screened DEPs that were clustered in the inflammatory response from the quantitative proteomics. We found 11 inflammation-related DEPs, comprising four upregulated DEPs (HP, ADIPOQ, VNN1, and LOC100517145) and seven downregulated DEPs (PRDX3, C1QTNF3, CHI3L1, RPS3, CALR, MMP8, and LTF) in our study. The GO and KEGG analyses showed that HP and VNN1 were enriched in the acute inflammatory response signaling pathway; ADIPPQ and C1QTNF3 were clustered in the negative regulation of inflammatory response signaling pathway; LOC100517145 and CHI3L1 were clustered in the inflammatory response signaling pathway; PRDX3, RPS3, and LTF were enriched in the positive regulation of NF-κB transcription factor activity signaling pathway; CALR and MMP8 were enriched in the positive regulation of NIK/NF-κB signaling. The inflammation-related DEPs are listed in **Table S4**.

### ASFV-infected PAMs upregulate the expression of the inflammatory factors and inhibit that of C1QTNF3

**Figure 4:**
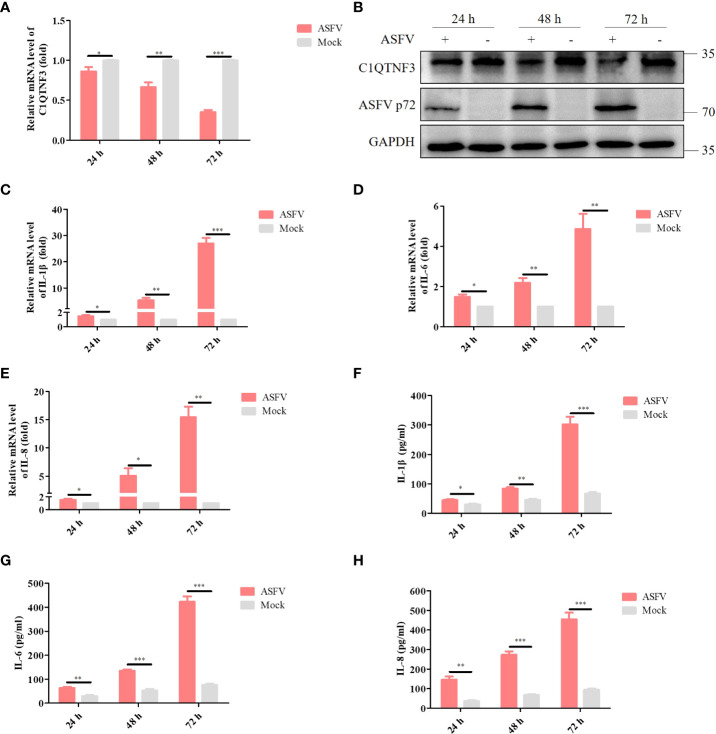
Expression of C1QTNF3 and pro-inflammatory factors IL-1β, IL-6, and IL-8 in ASFV-infected and mock-infected PAMs at 24, 48, and 72 h post-infection. The mRNA levels of **(A)** C1QTNF3, **(C)** IL-1β, **(D)** IL-6, and **(E)** IL-8 in ASFV-infected and mock-infected PAMs. The GAPDH gene was used as the internal control for normalization. The 2^-ΔΔCt^ method was used to analyze the results of qPCR. The protein levels of **(B)** C1QTNF3, ASFV p72 **(F)** IL-1β, **(G)** IL-6, and **(H)** IL-8 in ASFV-infected and mock-infected PAMs p <0.05 (*), p < 0.01 (**), and p < 0.001 (***).

The complement component is one of the important parts of serum samples, and regulates multiple signaling pathways, including inflammatory response (31). Therefore, we investigated the role of complement-related proteins in these inflammation-related DEPs, and found that among the 11 DEPs, C1QTNF3 is a member of the complement C1q/TNF-related protein family. Previous research has shown that ASFV infection could cause an imbalanced pro- and anti-inflammatory response (32). From the proteomics data, C1QTNF3 decreased by 0.591 times in ASFV-infected cells compared with that in the control. Previous research has indicated that C1QTNF3 regulates the inflammatory signaling pathway (33), and may be critical for ASFV-induced inflammatory response. Hence, we explored whether ASFV replication affects C1QTNF3 expression *in vitro*. PAMs were infected with ASFV at a multiplicity of infection (MOI) of 0.1, and the cell samples were collected 24 h, 48 h, and 72 h postinfection (hpi) to detect the expression of C1QTNF3 and other inflammatory factors. The results show that the expression of C1QTNF3 decreased gradually with infection time, which was 69% lower in the infected group than in the uninfected group at 72 hpi (**Figure 4A**). This result was consistent with that of the western blot analysis, and ASFV p72 proteins increased gradually with infection time (**Figure 4B**). The mRNA levels of the inflammatory factors, namely IL-1β, IL-6, and IL-8, increased gradually with the extended infection time (**Figures 4C-E**). Compared with the ASFV uninfected group, the levels of IL-1β, IL-6, and IL-8 in the ASFV-infected PAMs group increased by 26.9-, 4.86-, and 15.4-fold at 72 hpi, respectively. Simultaneously, the protein levels of the inflammatory factors, including IL-1β, IL-6, and IL-8, were upregulated comparing the ASFV-infected to uninfected with infection time (**Figures 4F-H**). These results show that the inflammatory responses were activated, and the expression of C1QTNF3 was inhibited in PAMs after ASFV infection.

### Knockdown of C1QTNF3 increases the expression of inflammatory cytokines and inhibits ASFV replication in PAMs

**Figure 5:**
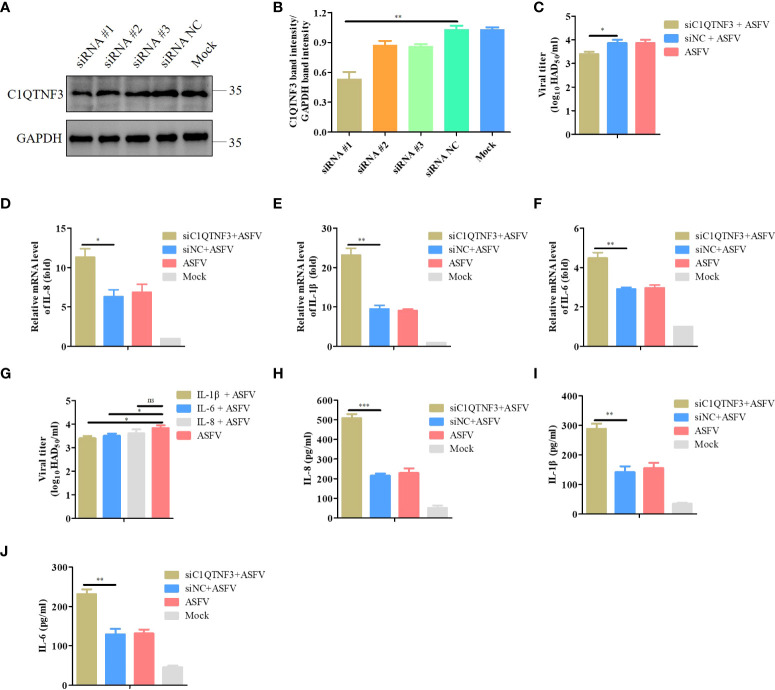
Upregulated pro-inflammatory cytokines inhibit ASFV infection in PAMs. **(A, B)** Detection of C1QTNF3 siRNA knockdown efficiency using western blot analysis. **(C)** C1QTNF3 knockdown in PAMs inhibits ASFV replication as detected using the HAD assay. The mRNA levels of **(D)** IL-8, **(E)** IL-1β, and **(F)** IL-6 upon C1QTNF3 knockdown in PAMs infected with ASFV. The GAPDH gene was used as the internal control for normalization. The 2^-ΔΔCt^ method was used to analyze the results of qPCR. **(G)** Porcine IL-1β, IL-6, and IL-8 proteins inhibit ASFV replication, as detected using the HAD assay. The protein levels of **(H)** IL-8, **(I)** IL-1β, and **(J)** IL-6 upon C1QTNF3 knockdown in PAMs infected with ASFV p <0.05 (*), p < 0.01 (**), and p < 0.001 (***), ns means no difference.

To determine whether C1QTNF3 affected the inflammatory response signaling pathway upon ASFV infection, we transfected siRNAs to downregulate the C1QTNF3 gene in PAMs. Western blot analysis results showed that among the several siRNAs, the siRNA #1 demonstrated the best inhibition efficiency (**Figure 5A**), which was consistent with the analysis of the relative protein band intensity using ImageJ software (**Figure 5B**). Hence, we chose the siRNA #1 for the subsequent experiments. Upon the downregulation of the C1QTNF3 gene by siRNA in PAMs 24 h before ASFV infection, the mRNA levels of IL-8, IL-1β, and IL-6 were upregulated in the C1QTNF3 downregulated cells compared with that in the siNC-treated cells at 48 hpi (**Figures 5D-F**). Compared with the siNC-treated cells followed by ASFV infection, the levels of IL-6, IL-1β, and IL-8 in the C1QTNF3 downregulated cells followed by ASFV infection increased by 1.5-, 2.4-, and 1.76-fold, respectively. The protein levels of IL-8, IL-1β, and IL-6 were also detected using ELISA (**Figures 5H-J**). The results were consistent with the qPCR results. In addition, the ASFV titer was lower in the ASFV-infected PAMs with C1QTNF3 downregulation by siRNA than in the siNC-treated PAMs (**Figure 5C**). To explore the role of inflammatory cytokines during ASFV proliferation, the cytotoxicity of porcine IL-1β, IL-6, and IL-8 proteins was measured in the PAMs using the CCK-8 assay according to the manufacturer’s instruction. When the concentrations of IL-1β, IL-6, and IL-8 proteins were less than 300 ng/mL, no cytotoxic effects were detected after 24 h or 48 h (**Figure S2**). Therefore, 50 ng/mL porcine IL-1β, IL-6, and IL-8 proteins were incubated with the PAMs for 24 h, followed by infection with 0.1 MOI ASFV for 48 h. The ASFV titer of the cell supernatants was measured using the HAD assay. The ASFV titer decreased in the PAMs treated with porcine IL-1β, IL-6, and IL-8 proteins compared with that of the untreated cells (**Figure 5G**). Thus, the downregulation of C1QTNF3 could lead to the upregulation of ASFV-induced inflammatory cytokines, and the expression of specific inflammatory cytokines can inhibit ASFV proliferation.

### Overexpression of C1QTNF3 inhibits the levels of inflammatory cytokines and benefits viral proliferation in PAMs

**Figure 6:**
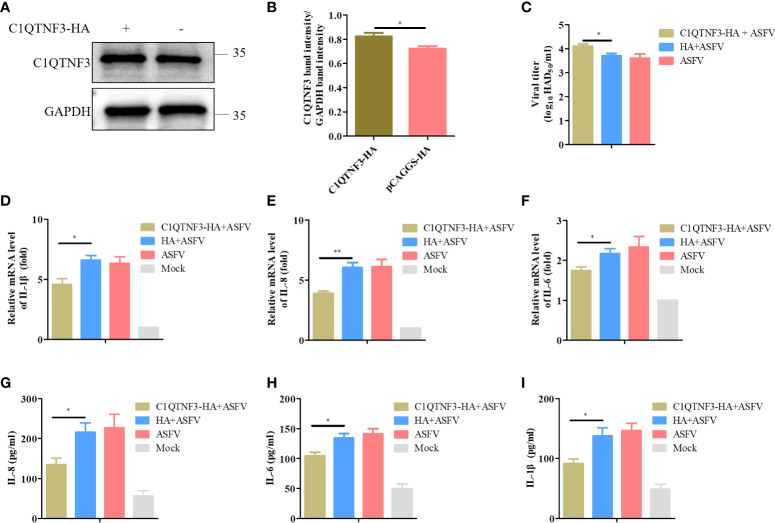
Downregulated pro-inflammatory cytokines increase ASFV infection in PAMs. **(A, B)** Overexpression of HA-tagged C1QTNF3 proteins was detected using western blot analysis. **(C)** C1QTNF3 overexpression in PAMs increases ASFV replication as detected using the HAD assay. The mRNA levels of **(D)** IL-1β, **(E)** IL-8, and **(F)** IL-6 upon C1QTNF3 overexpression in PAMs infected with ASFV. The GAPDH gene was used as the internal control for normalization. The 2^-ΔΔCt^ method was used to analyze the results of qPCR. The protein levels of **(G)** IL-8, **(H)** IL-6, and **(I)** IL-1β upon C1QTNF3 overexpression in PAMs infected with ASFV p <0.05 (*), p < 0.01 (**).

We explored the inflammatory response signaling pathway regulated by C1QTNF3 after ASFV infection in PAMs. PAMs were transfected with the C1QTNF3-HA plasmid for 24 h, followed by infection with ASFV for 48 h. The protein level of C1QTNF3 was detected using western blot analysis (**Figure 6A**), and the band intensity was analyzed using ImageJ (**Figure 6B**), which confirmed that C1QTNF3 was overexpressed in the PAMs. Comparing the C1QTNF3 overexpression group with the control group, the mRNA levels of IL-1β, IL-8, and IL-6 were reduced (**Figures 6D-F**). The protein levels of IL-8, IL-6, and IL-1β also showed concordant results (**Figures 6G-I**). The results indicate that the ASFV titer was higher in the supernatant of the ASFV-infected PAMs with C1QTNF3 overexpression than in the control group (**Figure 6C**). Therefore, C1QTNF3 overexpression could cause the downregulation of inflammatory cytokines after ASFV infection and further promoted viral proliferation.

### Overexpression of C1QTNF3 inhibits poly (dA:dT)-induced expression of inflammatory cytokines in PK-15

**Figure 7:**
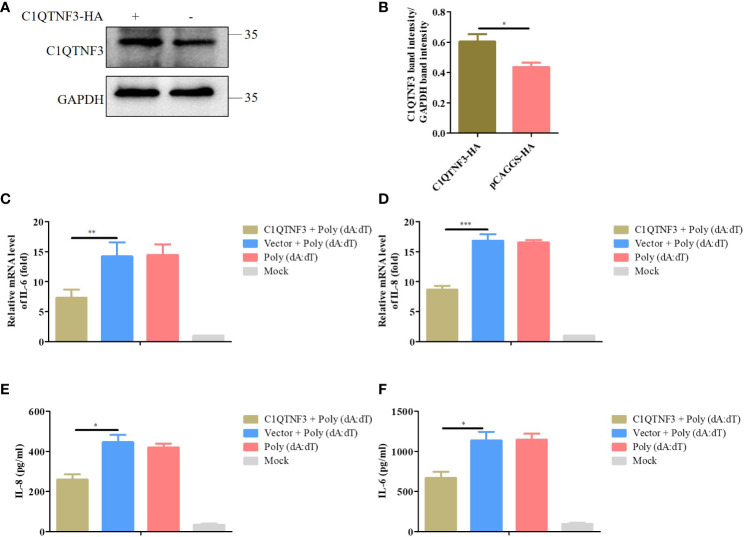
Overexpression C1QTNF3 inhibits poly (dA:dT)-induced inflammatory cytokine production in PK-15 cells. **(A, B)** Overexpression of HA-tagged C1QTNF3 proteins was detected using western blot analysis. The mRNA levels of **(C)** IL-6 and **(D)** IL-8 in PK-15 cells with C1QTNF3 overexpression for 24 h followed by poly (dA:dT) stimulation for 48 h. The GAPDH gene was used as the internal control for normalization. The 2^-ΔΔCt^ method was used to analyze the results of qPCR. The protein levels of **(E)** IL-8 and **(F)** IL-6 in PK-15 cells with C1QTNF3 overexpression for 24 h followed by poly (dA:dT) stimulation for 48 h p <0.05 (*), p < 0.01 (**), and p < 0.001 (***).

Considering the low transfection efficiency of PAMs, PK-15 cells were employed to assess the role of C1QTNF3 overexpression in the inflammatory signaling pathway. ASFV cannot infect PK-15 cells, and thus poly (dA:dT) double-stranded DNA fragments were selected as the inflammatory stimulus for these cells. The PK-15 cells were transfected with the C1QTNF3-HA plasmid for 24 h, followed by transfection with poly (dA:dT) for 48 h. The antibody of porcine endogenous C1QTNF3 was used to detect the protein level of overexpression of HA-tagged C1QTNF3 in the PK-15 cells using western blot analysis (**Figure 7A**), and the relative protein band intensity was analyzed using ImageJ software (**Figure 7B**), which show that C1QTNF3 was overexpressed in the PK-15 cells. Additionally, the mRNA level of TLR9 was upregulated comparing PK-15 cell transfection with poly (dA:dT) to the control cells, which confirmed that the inflammatory signaling pathway was activated (**Figure S3**). The mRNA levels of IL-6 and IL-8 were lower in cells with C1QTNF3 overexpression than in the control group (**Figures 7C, D**). The same results were also obtained for the protein levels of IL-8 and IL-6 (**Figures 7E, F**). Hence, it can be concluded that C1QTNF3 was highly likely to be a broad-spectrum anti-inflammatory mediator and is negatively associated with the expression of pro-inflammatory cytokines in an inflammatory reaction.

### Knockdown of C1QTNF3 increases poly (dA:dT)-induced expression of inflammatory cytokines in PK-15

**Figure 8:**
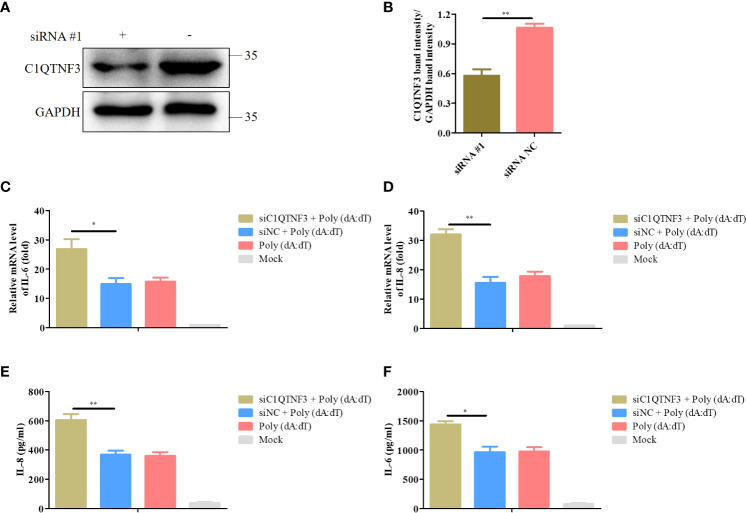
Knockdown of C1QTNF3 increases poly (dA:dT)-induced inflammatory cytokine production in PK-15 cells. **(A, B)** Detection of C1QTNF3 siRNA knockdown efficiency using western blot analysis. The mRNA levels of **(C)** IL-6 and **(D)** IL-8 in PK-15 cells with C1QTNF3 downregulation for 24 h followed by poly (dA:dT) stimulation for 48 h. The GAPDH gene was used as the internal control for normalization. The 2^-ΔΔCt^ method was used to analyze the results of qPCR. The protein levels of **(E)** IL-8 and **(F)** IL-6 in PK-15 cells with C1QTNF3 downregulation for 24 h followed by poly (dA:dT) stimulation for 48 h p <0.05 (*), p < 0.01 (**).

We explored the effect of the knockdown of C1QTNF3 in poly (dA:dT)-induced expression of inflammatory cytokines. PK-15 cells were transfected with siRNA #1 for 24 h, followed by transfection with poly (dA:dT) for 48 h. The antibody of porcine endogenous C1QTNF3 was used to detect the protein level of C1QTNF3 in the PK-15 cells using western blot analysis (**Figure 8A**), and the relative protein band intensity was analyzed using ImageJ software (**Figure 8B**), which indicates that C1QTNF3 was downregulated in the PK-15 cells. The mRNA levels of IL-6 and IL-8 were higher in the PK-15 cells with C1QTNF3 downregulation by siRNA #1 than in the siNC-treated PK-15 cells (**Figures 8C, D**). The same results were obtained for the protein levels of IL-8 and IL-6 (**Figures 8E, F**). Therefore, these results further indicate that the expression of C1QTNF3 was negatively correlated with that of the inflammatory cytokines.

## Discussion

A virus hijacks the host’s transcription and translation to complete its replication (34, 35). Simultaneously, the host cells develop several antiviral mechanisms after sensing the viral genetic material (36, 37). If there is an imbalance between the viral infection and host regulation, it may lead to cell death or virus cleaning. Therefore, exploring the host-virus interaction is critical for understanding the pathogenic mechanisms of the virus. In this study, to unravel the mechanisms of the host’s response to ASFV infection, we compared the serum protein level profiles between ASFV-infected samples and the uninfected. Upon label-free LC-MS/MS quantification, 173 DEPs were found between the two groups. These DEPs were involved in several biological processes, comprising the immune system, metabolism, and inflammation. Inflammatory response plays an important role in ASFV proliferation. The serum samples show the changes in multiple inflammatory cytokines after ASFV infection (32). Therefore, the proteomic analysis of serum samples can better reveal the inflammatory response after ASFV infection than other omics. However, the weakness of proteomics of serum samples is that the DEPs are mainly located in the extracellular region compared with the omics of cell or tissue samples, which leads to numerous intracellular proteins not being detected (13, 14). In the present study, C1QTNF3 was downregulated after ASFV infection, which increased the ASFV-induced inflammatory response, inhibiting ASFV replication.

Our data showed that natural and acquired immune responses were activated during ASFV infection (**Figures 2C, D**). ASFV has been reported to encode various proteins to repress the host’s immunity and complete its propagation. For instance, ASFV Armenia/07 inhibits the cGAS-STING pathway by suppressing STING activation, thereby restraining type I interferons and interferon-stimulated gene expression (38). ASFV E120R interferes with the recruitment of IRF3 to TANK-binding kinase 1, which suppresses IRF3 phosphorylation, decreasing interferon expression (39). Previous proteomic and transcriptomic studies have shown that the metabolic pathways are activated in ASFV-infected PAMs (12, 13). Fatty acid synthesis is essential for the red-spotted grouper nervous necrosis virus to complete its life cycle (40). In this study, we found that the upregulated proteins were involved in regulating fatty acid metabolism after ASFV infection. However, the metabolic pathway involved in ASFV infection remains to be further investigated, which will contribute to understanding the pathogenic mechanisms of ASFV.

ASFV infection activates an inflammatory response *in vivo* and *in vitro* (32, 41), which was corroborated by our KEGG analysis result that DEPs were enriched in the inflammatory signaling pathways (**Figure 3A**). The balance of inflammatory factor expression is vital for the host’s response to ASFV infection. C1QTNF3, as a multifunctional protein, is an anti-inflammatory mediator, and it negatively regulates the pro-inflammatory cytokines TNF-α, IL-6, and C-reactive protein (33). This ability may explain our finding that the expression of C1QTNF3 was negatively correlated with that of the inflammatory cytokines IL-6, IL-8, and IL-1β after ASFV infection (**Figure 4**). One previous study showed that miR-495-targeted C1QTNF3 inhibits hepatocellular carcinoma cell growth (42). In this study, the knockdown of the C1QTNF3 gene in ASFV-infected PAMs resulted in the increased expression of inflammatory cytokines, thereby inhibiting viral infection (**Figure 5**). Furthermore, we found that porcine IL-1β, IL-8, and IL-6 proteins repressed ASFV replication (**Figure 5G**). However, how C1QTNF3 regulates the inflammation process during ASFV infection needs further study.

Previous microarray analysis has identified C1QTNF3 as a downstream molecule of HIF-1α, and C1QTNF3 exerts anticatabolic effects by suppressing NF-κB (43). The inflammatory response is reported to resist viral proliferation; for instance, ASFV MGF505-7R inhibits NF-κB activation and NLRP3 inflammasome assembly, thus reducing IL-1β production, finally promoting viral replication (26). ASFV-encoded early viral protein UBCv1 can block p65 nuclear translocation, inhibiting the inflammatory response (44). I226L, A151R, NP419L, and QP383R also inhibit the inflammatory response in an iGLuc reporter system (45). Moreover, the human cytomegalovirus-encoded protein IE86 impairs the synthesis of the IL-1β transcript and the stability of the immature protein, thus leading to immune evasion through the regulation of inflammasome activation (46). We found that in ASFV-infected PAMs, the expression of inflammatory cytokines was upregulated, and the C1QTNF3 expression was downregulated. Overexpression of C1QTNF3 inhibited inflammatory cytokine production after ASFV infection, in turn enhancing viral proliferation (**Figure 6**). However, whether the interaction between C1QTNF3 and ASFV-encoded proteins inhibiting the inflammatory response regulates inflammation and viral proliferation deserves further investigation. In addition, the overexpression of C1QTNF3 could inhibit the production of poly (dA:dT)-induced inflammatory cytokines (**Figure 7**). Therefore, C1QTNF3 may be a broad-spectrum anti-inflammatory mediator and a potential drug to reduce an inflammatory reaction.

In conclusion, this study revealed the ASFV-host interactions in ASFV-infected and uninfected serum samples through quantitative proteomics. We also found that reducing C1QTNF3 expression promotes the production of ASFV infection-induced inflammatory cytokines, thereby inhibiting viral replication. Therefore, this study provides insights into potential antiviral targets in the pathogenic process of ASFV infection.

## Data availability statement

The datasets presented in this study can be found in online repositories. The names of the repository/repositories and accession number(s) can be found below: http://www.proteomexchange.org, accession ID: PXD035545.

## Ethics statement

The animal study was reviewed and approved by Research Ethics Committee, Huazhong Agricultural University, Hubei, China.

## Author contributions

CJL, MJ, and XL contributed to conception and design of the study. QZ and ZZ organized the database. CFL and XS performed the statistical analysis. CJL wrote the first draft of the manuscript. LZ, JY, and YZ wrote sections of the manuscript. All authors contributed to manuscript revision, read, and approved the submitted version.

## Funding

This study was supported by the National Key R&D Program of China (Approval No: 2021YFD1801405) and Key technologies for ASFV control from the Department of Science and Technology of Hubei province, China (Approval No: 2019ABA08).

## Acknowledgments

We would like to thank the National Key R&D Program of China, the Department of Science and Technology of Hubei province, and all the members of the animal biosafety level 3 (ABSL-3) laboratory of Huazhong Agricultural University.

## Conflict of interest

Authors CL, ZZ and MJ were employed by company Wuhan Keqian Biology Co., Ltd.

The remaining authors declare that the research was conducted in the absence of any commercial or financial relationships that could be construed as a potential conflict of interest.

## Publisher’s note

All claims expressed in this article are solely those of the authors and do not necessarily represent those of their affiliated organizations, or those of the publisher, the editors and the reviewers. Any product that may be evaluated in this article, or claim that may be made by its manufacturer, is not guaranteed or endorsed by the publisher.
