# Supplementary material for: African swine fever virus infection activates inflammatory responses through downregulation of the anti-inflammatory molecule C1QTNF3
Source: Front Immunol. 2022 Oct 12;13:1002616. doi: 10.3389/fimmu.2022.1002616 (PMC9598424; doi:10.3389/fimmu.2022.1002616)
Supplement: Supplementary file 1 [file DataSheet_1.pdf]

Supplementary Materials

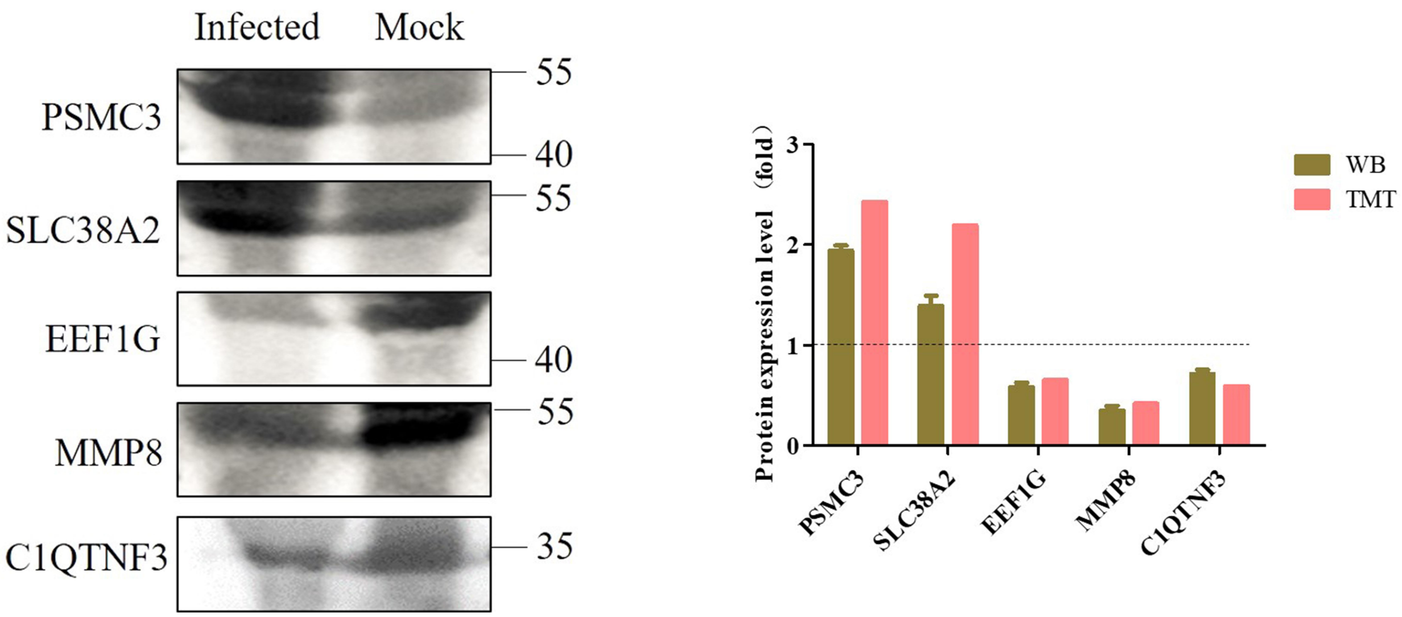

Supplementary Figure 1. Western blot analysis of the ASFV-infected and un-infected serum samples.

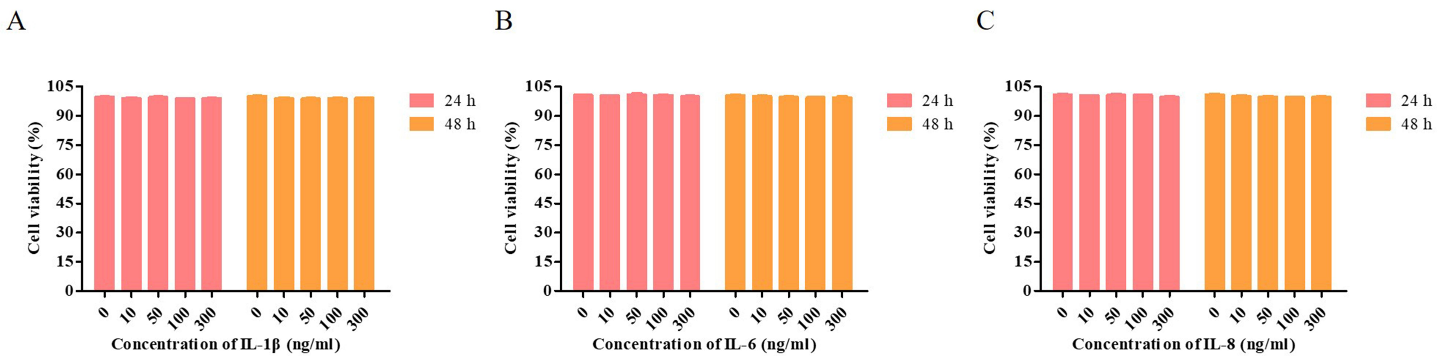

Supplementary Figure 2. Analysis of the cytotoxicity of porcine IL-1β (A), IL-6 (B), and IL-8 (C) protein to PAMs after 24 h and 48 h treatments by the CCK-8 assay.

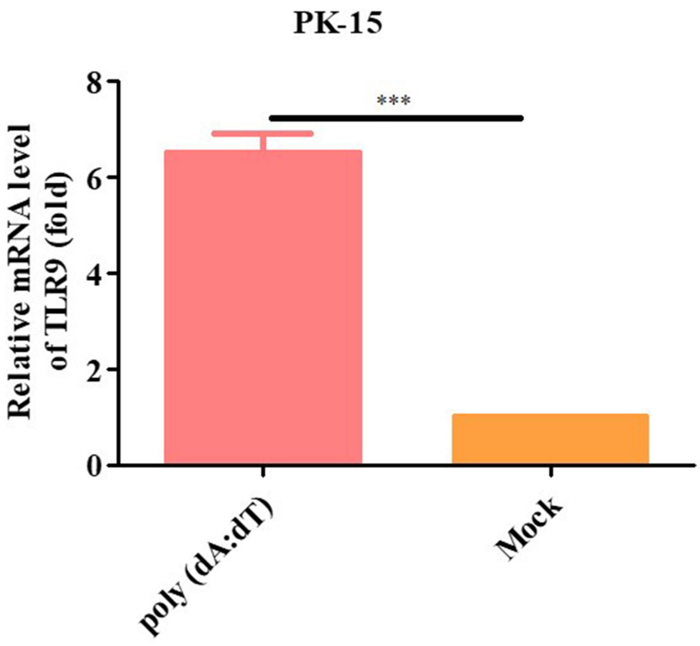

Supplementary Figure 3. The mRNA expression level of TLR9 in PK-15 cells following by poly (dA:dT) transfection.
